# An improved discriminative filter bank selection approach for motor imagery EEG signal classification using mutual information

**DOI:** 10.1186/s12859-017-1964-6

**Published:** 2017-12-28

**Authors:** Shiu Kumar, Alok Sharma, Tatsuhiko Tsunoda

**Affiliations:** 1 Department of Electronics, Instrumentation and Control Engineering, School of Electrical & Electronics Engineering, Fiji National University, Suva, Fiji; 2 School of Engineering and Physics, Faculty of Science, Technology and Environment, The University of the South Pacific, Suva, Fiji; 3 Institute for Integrated and Intelligent Systems (IIIS), Griffith University, Brisbane, Australia; 4 RIKEN Center for Integrative Medical Sciences, Yokohama, 230-0045 Japan; 5 CREST, JST, Yokohama, 230-0045 Japan; 6 Department of Medical Science Mathematics, Medical Research Institute, Tokyo Medical and Dental University (TMDU), Tokyo, 113-8510 Japan

**Keywords:** Brain computer interface, Common spatial pattern, Electroencephalography, Frequency band, Motor imagery, Mutual information

## Abstract

**Background:**

Common spatial pattern (CSP) has been an effective technique for feature extraction in electroencephalography (EEG) based brain computer interfaces (BCIs). However, motor imagery EEG signal feature extraction using CSP generally depends on the selection of the frequency bands to a great extent.

**Methods:**

In this study, we propose a mutual information based frequency band selection approach. The idea of the proposed method is to utilize the information from all the available channels for effectively selecting the most discriminative filter banks. CSP features are extracted from multiple overlapping sub-bands. An additional sub-band has been introduced that cover the wide frequency band (7–30 Hz) and two different types of features are extracted using CSP and common spatio-spectral pattern techniques, respectively. Mutual information is then computed from the extracted features of each of these bands and the top filter banks are selected for further processing. Linear discriminant analysis is applied to the features extracted from each of the filter banks. The scores are fused together, and classification is done using support vector machine.

**Results:**

The proposed method is evaluated using BCI Competition III dataset IVa, BCI Competition IV dataset I and BCI Competition IV dataset IIb, and it outperformed all other competing methods achieving the lowest misclassification rate and the highest kappa coefficient on all three datasets.

**Conclusions:**

Introducing a wide sub-band and using mutual information for selecting the most discriminative sub-bands, the proposed method shows improvement in motor imagery EEG signal classification.

## Background

Communication is the transfer of information through various ways such as speaking, writing, using sign language or other mediums, and is essential in our daily lives. Human brain is one of the key parts of the human body controlling all the body activities including motor and muscle movement. Every time a communication is initiated, the message is first constructed in the brain. Over 100 billion neurons are contained by the human brain [1]. These neurons communicate with each other producing different patterns of electrical signals (generated due to electromagnetic activities inside the brain) for different thoughts [2]. These electrical signals are known as the electroencephalography (EEG) signals. The purpose of a brain computer interface (BCI) system is to capture the EEG signal and decode them for different brain activities. This provides the brain a direct channel of communication with the external devices without the need for any muscular movement [3].

Over the past two decades, advances in signal processing, pattern recognition and machine learning techniques have resulted in a great progress for BCI research [4]. A huge amount of focus is dedicated to the field of biomedical engineering [5–16], with focus on BCI research. The severely disabled people can benefit from the BCI system to reinstate their ability of environmental control [17]. BCI has several applications such as communication control [18, 19], environment control [20], movement control [21, 22] and neuro-rehabilitation [23–25]. The use of non-invasive EEG sensors to capture the EEG signal has gained widespread attention out of the many other available methods. This is because non-invasive EEG devices such as Emotiv EPOC/EPOC+ headset [26] is portable, can be easily integrated for real time analysis and has comparatively low cost. Thus, it is the most suitable method to capture EEG signals for BCI systems [27, 28]. The EEG signal captures all the activities that are taking place in the brain and thus it is referred to as a complex signal. The raw EEG signal is a weak signal with very low amplitudes and is generally contaminated by artifacts and noise such as Electrocardiogram (ECG), Electrooculogram (EOG) and Electromyogram (EMG). Therefore, preprocessing of the raw EEG signals is mostly carried out to remove artifacts and noise.

EEG signals can be grouped into different frequency bands as different type of information is contained in different bands. Various methods of feature extraction and classification [13–15, 29–31] have been proposed. CSP has been most superior and widely used feature extraction method. CSP transforms the data to a new time series where the variance of one class of signal is maximized and that of another class is minimized. However, feature extraction of motor imagery EEG signal using CSP hugely depends on the selection of the frequency bands. Since the frequency bands are subject-specific, it is difficult to determine the optimal filter bands. Poorly selected bands will mostly not be able to capture the band-power changes that the motor imagery event causes resulting in CSP being less effective [32]. Generally, a wide band (eg., 4–40 Hz) is selected for CSP in motor imagery EEG signal classification. This wide band covered most of the motor imagery related features, however, it also contained other redundant information. Over the past few years, studies [13, 32–37] have suggested that optimizing the filter band could improve the motor imagery EEG signal classification. Common spatio-spectral pattern (CSSP) [38] has been proposed to further enhance the performance of CSP. In CSSP, a finite impulse response (FIR) filter is optimized within CSP. This is realized by inserting a temporal delay *τ* allowing frequency filters to be tuned individually and CSSP achieved improved performance. Common sparse spectral spatial pattern (CSSSP) [39] was proposed to further improve the CSSP approach, which finds spectral patterns that is common to all the channels instead of finding different spectral patterns for each channel as in CSSP.

**Fig. 1:**
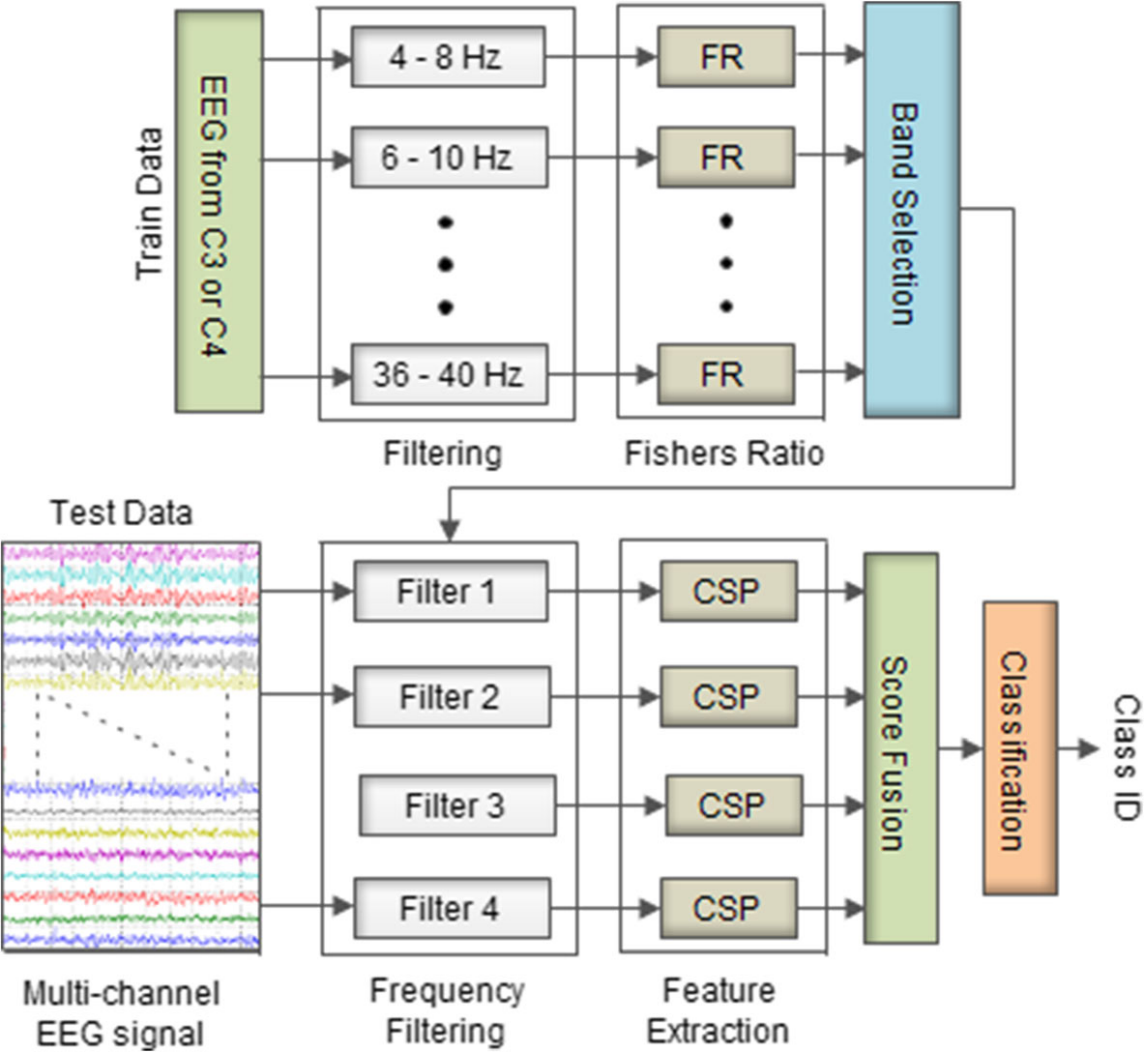
The DFBCSP framework

As an alternative method, sub-band common spatial pattern (SBCSP) [40] has been proposed, where the motor imagery EEG signals are filtered at multiple sub-bands and CSP features are extracted from each of the sub-bands. To reduce the dimensionality of the sub-bands linear discriminant analysis (LDA) has been applied separately to the features of each of the sub-bands and the scores fused together for classification. SBCSP achieved superior classification accuracy than those of CSP, CSSP and CSSSP. However, the possible association of the CSP features obtained from different sub-bands has been ignored by SBCSP and therefore filter bank CSP (FBCSP) [32] was proposed to address this problem. FBCSP estimates the mutual information of the CSP features from multiple sub-bands in order to select the most discriminative features. The selected features are used for classification using support vector machine (SVM) classifier. FBCSP outperformed SBCSP, however, it still utilized several sub-bands that accounts for an increased computational cost. Discriminant filter bank CSP (DFBCSP) [35, 36] has been proposed to address this problem. DFBCSP utilizes the fisher ratio (FR) of single channels (C3, C4 or Cz) band power for selecting the most discriminant sub-bands from multiple overlapping sub-bands. The CSP features are then extracted for each sub-band, and used for classification using SVM classifier. DFBCSP achieved improved classification accuracy and a reduced computational cost compared to SBCSP and FBCSP. The DFBCSP framework is shown in Fig. 1.

In CSP, empirical averaging of training samples covariance matrices is done. This includes the low quality signals, which degrades the performance of the system. Therefore, the authors in [41] proposed a sparsity-aware method where weighted averaging has been introduced. Using *l*_1_ minimization problem, weight coefficients are assigned to each of the trials. The low quality trials get assigned to almost zero weight values. This weighting method was applied for determining the average covariance matrix in the CSP algorithm and it achieved improved performance. In [30], the authors proposed to use decimation filter that was manually tuned to obtain optimal results. Fishers’ discriminant analysis (FDA) was used to reduce the dimensionality of the features and SVM classifier was employed. The method (named CD-CSP-FDA) achieved improved performance compared to the state-of-the-art methods.

Recently, a sparse filter bank CSP (SFBCSP) [42] method that also uses multiple filter bands is proposed, which optimizes the sparse patterns. Supervised technique is used to select significant CSP features from multiple overlapping frequency bands. SVM classifier is then used for motor imagery classification using the selected features. Sparse Bayesian learning has also gained increased attention recently and has been used for feature selection in various applications. In [13], the EEG signal was decomposed into multiple sub-bands and CSP features were extracted. Sparse features are obtained using the Bayesian learning approach, which are used for classification using the SVM classifier. The authors named their method as SBLFB and it outperformed all the state-of-the-art methods. In [43] a hybrid genetic algorithm-particle swarm optimization based means clustering has been proposed for 2 class motor imagery tasks. However, clustering methods [44, 45] and hidden markov model [46] have not been fully explored for motor imagery EEG signal classification.

In this paper, we propose an improved DFBCSP method. The contribution and novelty of the proposed approach, which makes our proposed approach different from DFBCSP method are as follows. Firstly, instead of using FR of single channels band power as in DFBCSP-FR, we use mutual information calculated from features generated using all channel data for selecting the bands that give optimal results. Using only a single channels band power with FR as the criterion for selecting the sub-bands (DFBCSP-FR) will not be effective. This is due to the fact that EEG signals are mostly contaminated by noise. Therefore, if the single channel used for calculating FR is corrupted by noise, then this band selection method will fail. This results in sub-bands being selected that will not always give optimal results as sub-bands with redundant information might be selected. Thus, we propose to utilize all available channels data for selecting the most discriminant sub-bands by making use of the mutual information in order to obtain optimal results. Using all channels data for band selection reduces the chance of a sub-band with redundant information being selected compared to that of using single channel information for band selection.

Secondly, instead of using only CSP features from overlapping sub-bands as in DFBCSP-FR, we have introduced an additional wide band of 7–30 Hz with CSP and CSSP features. In our previous work [30], we have shown that promising results can be obtained by using a single wide band in the frequency range of 7–30 Hz. It is also shown that using wide band CSP and CSSP methods produce promising results for some subjects (refer to Table 1, Table 2 and Table 3) that other competing methods could not achieve. Therefore, to take advantage of the wide band CSP and CSSP, we have introduced a single wide band of 7–30 Hz together with the twelve overlapping sub-bands in the range of 4–30 Hz having a bandwidth of 4 Hz and overlap of 2 Hz. Both CSP and CSSP features are extracted from the wide band. Use of the CSP and CSSP features of the wide band boosts the performance of the system in majority cases by providing features that are more significant (making it to the top 4 sub-bands having most discriminant features). Thus, the sub-bands with more significant information are selected, and optimal results are achieved. This is shown by the reduction in the misclassification rate that is achieved, which is due to the fact that the wide band contains more significant information in majority cases (refer to Table 4, Table 5 and Table 6, which shows that the wide band is selected majority of the times).

**Table 1:** Misclassification rate (%) of different methods using dataset 1

Subject	CSP	CSSP	FBCSP	DFBCSP (FR)	DFBCSP (MI)	SFBCSP	SBLFB	Proposed
*aa*	21.00 ± 5.31	17.00 ± 7.34	17.14 ± 8.19	9.64 ± 5.01	11.50 ± 6.42	18.43 ± 7.45	18.71 ± 7.45	**8.79** ± 5.16
*al*	3.86 ± 3.63	3.07 ± 3.03	1.29 ± 1.18	**1.00** ± 1.91	1.21 ± 1.16	1.64 ± 1.36	1.36 ± 1.23	1.14 ± 1.03
*av*	28.29 ± 7.46	28.86 ± 7.10	30.36 ± 8.23	31.21 ± 8.92	25.28 ± 8.77	29.93 ± 6.44	29.64 ± 9.98	**24.05** ± 8.29
*aw*	10.36 ± 5.10	8.43 ± 5.09	6.50 ± 4.55	4.64 ± 4.75	3.93 ± 4.03	9.29 ± 5.85	6.57 ± 4.47	**3.21** ± 3.13
*ay*	**3.86** ± 4.11	4.29 ± 3.75	5.07 ± 4.68	8.21 ± 5.06	6.93 ± 4.47	12.79 ± 5.96	12.36 ± 7.22	4.43 ± 3.50
Average	13.47 ± 5.18	12.33 ± 5.30	12.07 ± 5.51	10.94 ± 5.13	9.77 ± 5.11	14.14 ± 5.57	13.73 ± 6.23	**8.32** ± 4.48

The lowest misclassification rate for each subject is indicated in bold

**Table 2:** Misclassification rate (%) of different methods using dataset 2

Subject	CSP	CSSP	FBCSP	DFBCSP (FR)	DFBCSP (MI)	SFBCSP	SBLFB	Proposed
*a*	**13.20** ± 8.07	13.65 ± 8.19	19.10 ± 9.35	16.80 ± 7.81	14.40 ± 5.68	17.40 ± 5.93	19.10 ± 9.73	14.30 ± 9.26
*b*	42.80 ± 12.25	42.70 ± 11.38	44.70 ± 11.27	42.90 ± 9.75	43.00 ± 9.69	45.30 ± 6.59	**41.50** ± 11.12	43.00 ± 10.74
*c*	43.70 ± 11.24	39.95 ± 10.21	35.70 ± 9.58	35.20 ± 8.51	33.70 ± 9.99	43.00 ± 11.62	33.20 ± 12.53	**31.00** ± 9.85
*d*	22.40 ± 8.82	14.60 ± 8.75	22.20 ± 8.99	23.50 ± 8.41	21.90 ± 8.59	29.50 ± 10.13	11.50 ± 7.91	**6.60** ± 5.57
*e*	18.00 ± 9.74	18.05 ± 9.18	14.00 ± 9.15	18.30 ± 8.84	17.30 ± 8.88	24.70 ± 10.34	11.60 ± 6.88	**8.10** ± 6.92
*f*	22.50 ± 10.84	18.55 ± 8.39	19.60 ± 8.56	14.30 ± 8.57	**13.00** ± 8.08	20.90 ± 6.45	21.20 ± 11.98	13.40 ± 8.48
*g*	7.10 ± 5.06	6.35 ± 4.92	6.90 ± 6.62	9.00 ± 5.05	7.60 ± 5.65	9.70 ± 4.97	**5.90** ± 5.41	7.20 ± 5.26
Average	24.24 ± 9.43	21.98 ± 8.72	23.17 ± 9.07	22.86 ± 8.13	21.56 ± 8.12	27.21 ± 8.00	20.57 ± 9.36	**17.66** ± 8.01

The lowest misclassification rate for each subject is indicated in bold

**Table 3:** Misclassification rate (%) of different methods using dataset 3

Subject	CSP	CSSP	FBCSP	DFBCSP (FR)	DFBCSP (MI)	SFBCSP	SBLFB	Proposed
*B0103T*	23.69 ± 10.37	25.31 ± 9.99	**19.00** ± 8.47	23.25 ± 11.23	20.38 ± 9.18	26.50 ± 9.24	21.75 ± 9.96	19.25 ± 10.48
*B0203T*	41.00 ± 11.21	42.94 ± 11.74	45.63 ± 11.93	40.76 ± 12.45	44.38 ± 11.24	42.75 ± 12.84	**40.75** ± 11.99	41.63 ± 10.23
*B0303T*	49.63 ± 10.80	48.44 ± 10.82	49.13 ± 13.54	50.50 ± 12.87	46.38 ± 9.95	44.97 ± 11.65	50.68 ± 13.34	**44.00** ± 13.06
*B0403T*	0.63 ± 0.60	0.63 ± 0.60	1.75 ± 1.61	0.75 ± 0.69	0.63 ± 0.60	**0.38** ± 0.35	0.88 ± 0.73	0.63 ± 0.60
*B0503T*	16.56 ± 9.21	42.25 ± 16.33	28.50 ± 8.85	25.00 ± 10.71	21.13 ± 9.36	25.02 ± 7.38	**7.96** ± 6.52	9.42 ± 7.96
*B0603T*	21.19 ± 9.89	23.81 ± 10.94	24.38 ± 9.80	20.88 ± 10.38	19.75 ± 9.81	20.06 ± 10.70	20.51 ± 8.23	**18.00** ± 9.91
*B0703T*	14.13 ± 8.46	13.81 ± 8.11	15.50 ± 6.83	12.13 ± 9.05	9.75 ± 7.05	12.25 ± 7.47	**7.50** ± 6.44	11.13 ± 7.61
*B0803T*	11.69 ± 7.14	14.50 ± 8.56	18.88 ± 11.68	11.13 ± 6.95	12.88 ± 8.03	12.38 ± 7.63	11.13 ± 8.95	**10.50** ± 5.85
*B0903T*	17.25 ± 8.15	17.25 ± 8.66	20.88 ± 10.07	22.25 ± 10.80	16.34 ± 8.93	25.00 ± 9.62	19.38 ± 10.58	**16.25** ± 9.36
Average	21.75 ± 8.57	25.44 ± 9.67	24.85 ± 9.39	22.96 ± 9.61	21.29 ± 8.38	23.26 ± 8.67	20.06 ± 8.73	**18.98** ± 8.48

The lowest misclassification rate for each subject is indicated in bold

**Table 4:** Top 4 bands mostly selected by the proposed method using dataset 1

Subject	*aa*	*al*	*av*	*aw*	*ay*
Selected bands	4, 5, 10, 11	4, 5, 13a, 13b	3, 4, 8, 13b	3, 4, 5, 13a	3, 4, 13a, 13b

**Table 5:** Top 4 bands mostly selected by the proposed method using dataset 2

Subject	*a*	*b*	*c*	*d*	*e*	*f*	*g*
Selected bands	3, 4, 13a,13b	4, 7, 8, 11	4, 5, 11, 13b	4, 5, 10, 13b	4, 5, 10, 13b	3, 4, 13a, 13b	2, 3, 8, 13b

**Table 6:** Top 4 bands mostly selected by the proposed method using dataset 3

Subject	*B0103T*	*B0203T*	*B0303T*	*B0403T*	*B0503T*	*B0603T*	*B0703T*	*B0803T*	*B0903T*
Selected bands	8, 9, 13a, 13b	1, 3, 4, 13a	1, 3, 4, 13a	3, 4, 13a, 13b	4, 10, 11, 13a	3, 4, 5, 13b	4, 5, 13a, 13b	3, 4, 13a, 13b	4, 10, 13a, 13b

The public BCI Competition III dataset IVa, BCI Competition IV dataset I and BCI Competition IV dataset IIb are used to validate the effectiveness of the proposed method in comparison with CSP, CSSP, FBCSP, DFBCSP, SFBCSP and SBLFB methods. Experimental results obtained are promising and can be instrumental in developing improved motor imagery based BCI systems.

## Methods

### Feature extraction using CSP

EEG based BCI has recently gained widespread attention in becoming a medium of communication between the human brain and the external world. CSP has been commonly used for feature extraction in EEG based BCI research and applications. In CSP, the spatial filter *W*_*csp*_ is formed by selecting the first and last *m* columns of the CSP matrix, ***W***. Thus, the bandpass filtered EEG signal *X*_*n*_ ∈*R* ^*C* x *T*^ is transformed using (1), where *n* denotes the *n*-th trial, c is the number of channels and t is the number of sample points.

1 $$ {Z}_n={W}_{CSP}^T\ {X}_n $$

The CSP features of *n*-th sample is then extracted using (2), where ${f}_n^i$ is the *i-*th feature of the *n-*th trial, and var(${Z}_n^j$) denotes the variance of *j-*th row of *Z*_*n*_. The feature matrix is thus formed as *F* = [*f*_1_; …; *f*_*N*_], where N is the total number of trials. A comprehensive explanation of CSP process can be obtained from [47].

2 $$ {f}_n^i=\log \left(\frac{\operatorname{var}\left({Z}_n^i\right)}{\sum_{j=1}^{2m}\operatorname{var}\left({Z}_n^j\right)}\right) $$

### Feature extraction using CSSP

The CSSP method was proposed in order to improve the performance of CSP by inserting a temporal delay to the raw signal. The time delay *τ* value of 1 to 15 sample points have been evaluated and the best value is selected using 10 fold cross validation. The signal is filtered using the bandpass filter followed by spatial filtering using (1) and feature extraction using (2).

### The improved DFBCSP approach

**Fig. 2:**
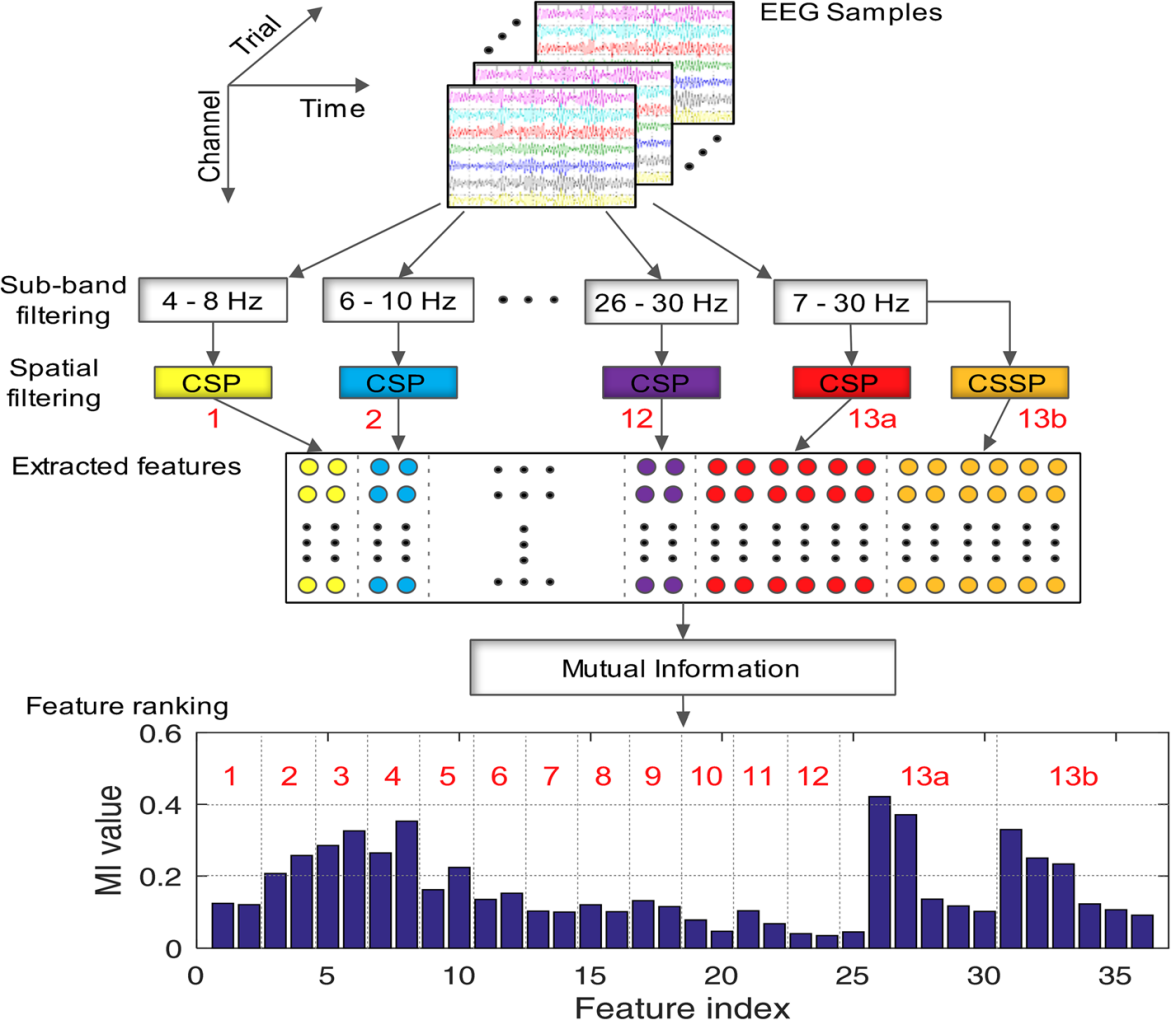
Illustration of calibration phase of the proposed approach (MI value - mutual information value of features of corresponding sub-bands indicated in red)

In this study, we propose an improved method that utilizes the mutual information for selecting the most discriminant filter banks (sub-bands) for motor imagery EEG signal classification. An illustration of the calibration phase of the proposed approach is given in Fig. 2. The dataset is divided into train and test data. Only train data is used in the calibration phase for selecting the filter banks. The train data is filtered using 13 filter banks. 12 filter banks are in the range of 4–30 Hz having a bandwidth of 4 Hz with 2 Hz overlap, and the final filter bank of 7–30 Hz.

**Fig. 3:**
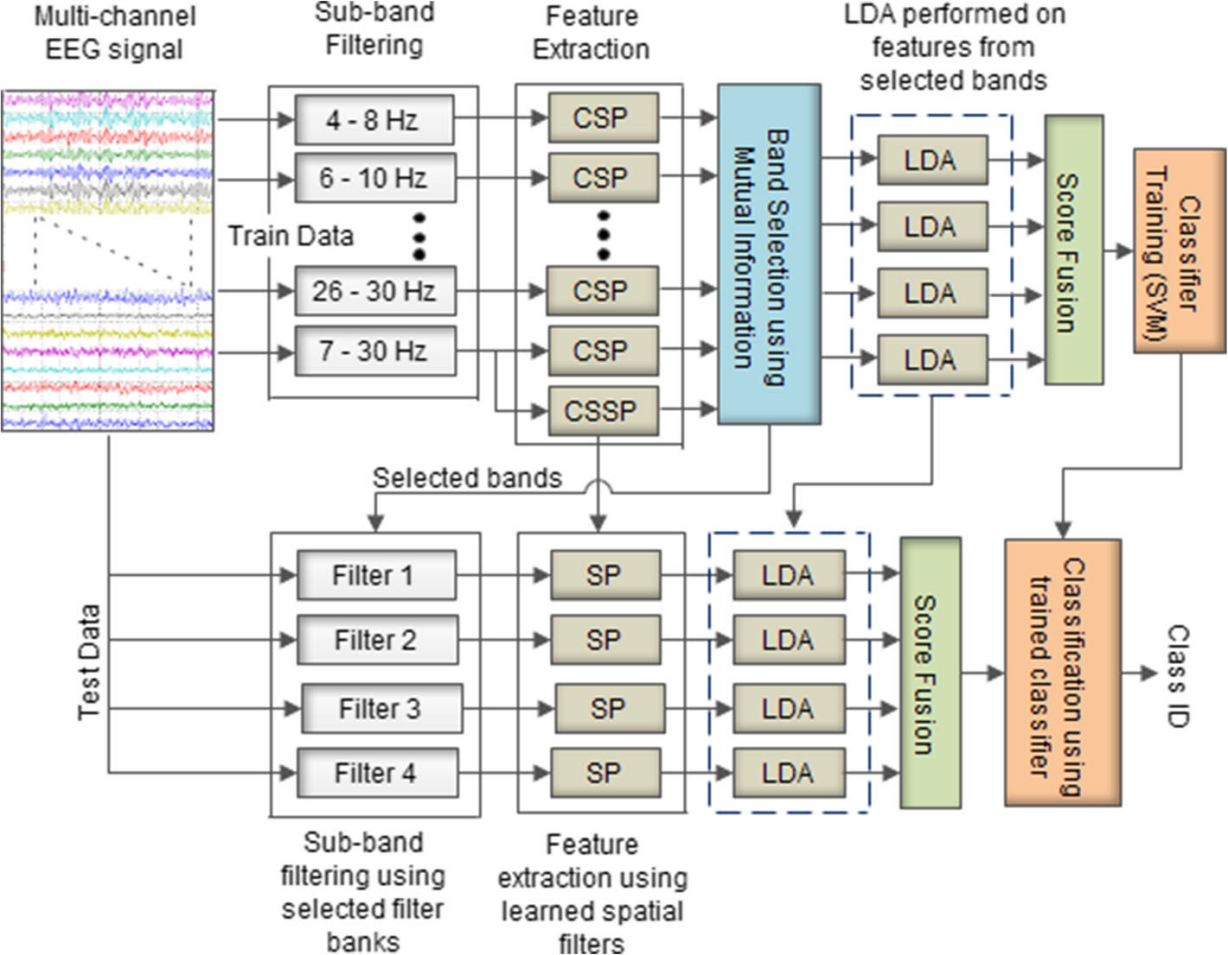
General framework of the proposed approach

Figure 3 shows the general framework of the proposed approach, giving detailed information for each of the steps. The raw EEG signals are decomposed into sub-bands, and CSP and CSSP features are extracted, respectively as shown in Fig. 3. Mutual information is then calculated from the feature matrix (refer to next sub-section) in order to determine the 4 most discriminating filter banks (filtered EEG signals of the filter banks that have more discriminating features, that is features with larger mutual information values). The maximum mutual information values for each of the sub-bands are used to form vector V_*MI*_ (having vector length of 14 since we have 14 sub-bands in total). The mutual information values in V_*MI*_ are arranged in descending order and the 4 bands to which the first 4 mutual information values in vector V_*MI*_ belong to are thus selected as the top 4 bands. The dimensionality of the features of each of the selected filter banks is reduced using linear discriminant analysis (LDA). The LDA scores are then fused together and fed to the SVM classifier. All parameters such as the filter banks, spatial filters, LDA matrix and the classifier are learned from the training data only and later used during the test phase.

### Mutual information

The quantity of information a feature contains about the class membership under the assumption of independence is given by the mutual information (MI). It is one of the measures of association or correlation between the row and column variables. The correlation coefficient only measures the linear dependence whereas mutual information gives information about both linear and non-linear dependence. For two discrete arbitrary variables *X* and *Y*, the mutual information can be computed using (3), where *p*(*x*,*y*) is the joint probability distribution function of *X* and *Y*, and *p*(*x*) and *p*(*y*) are the marginal probability distribution functions of *X* and *Y*, respectively. A larger mutual information value implies the corresponding feature has a greater predictive ability of the class membership (i.e. discriminating features).

Alternatively, the mutual information can also be computed using (4), where *H*(*Y*) is the marginal entropy, *H*(*X*| *Y*) and *H*(*Y*| *X*) are the conditional entropies and *H*(*X*, *Y*) is the joint entropy of *X* and *Y*.

3 $$ I\left(X,Y\right)={\sum \limits}_{y\in Y}{\sum \limits}_{x\in X}p\left(x,y\right)\log \left(\frac{p\left(x,y\right)}{p(x)p(y)}\right) $$

4 $$ I\left(X,Y\right)=H(Y)-H\left(Y|X\right)=H\left(X,Y\right)-H\left(X|Y\right)-H\left(Y|X\right) $$

The features obtained from all the bands are concatenated to form the feature vector ${F}_V^i=\left[{f}_{B1}^i,{f}_{B2}^i,\dots, {f}_{Bn}^i\right]$, where ${F}_V^i$ is the feature vector of the *i*-th trial, ${f}_{Bj}^i$ is the features obtained from the *j*-th band of the *i*-th trial, and *n* is the total number of bands. The feature matrix ${F}_M=\left[{F}_V^1;{F}_V^2;\dots; {F}_V^n\right]$, is formed using the feature vectors of all the trials from the train data. The feature matrix is then utilized to determine the mutual information using (3), which gives *MI* = [*I*_1_, *I*_2_, …, *I*_*L*_], where *I*_*l*_ is the mutual information value of the *l*-th feature.

## Experimental study

### Description of dataset

The proposed method has been evaluated using three publicly available datasets: BCI Competition III dataset IVa [48], BCI Competition IV dataset I [49] and, BCI Competition IV dataset IIb [49] referred to as dataset 1, dataset 2 and dataset 3 from here onwards, respectively.

Dataset 1 contains 118 channels of EEG signals for right hand and left foot MI tasks, which have been recorded from five subjects labeled *aa*, *al*, *av.*, *aw*, and *ay*. The down sampled signal at 100 Hz has been used. It contains 140 trials of each task for each of the subjects. A detail description of the dataset can be found online at http://www.bbci.de/competition/iii/.

Dataset 2 contains two classes of motor imagery EEG signals obtained from seven different subjects; 59 channels of data are recorded at 1000 Hz using BrainAmp MR plus amplifiers and Ag/AgCl electrode cap. The data were filtered using 10th order Chebyshev Type II lowpass filter with stopband ripple of 50 dB and stopband edge frequency of 49 Hz. The data was down sampled to 100 Hz by computing the mean of blocks of 10 samples. A total of 200 trials of motor imagery EEG measurements are available for each subject with almost equal number of trials for each class. A detailed description of the dataset can be found online at http://www.bbci.de/competition/iv/.

Dataset 3 contains 3 channels (C3, Cz, and C4) data for right hand and left hand motor imagery tasks recorded from nine subjects. The data was recorded at a sampling rate of 250 Hz. As in [42], only the third session data is used for evaluation. For each subject, a total of 160 trials of motor imagery EEG measurements are available (having equal number of trials for each motor imagery tasks). More details about the dataset can be found online at http://www.bbci.de/competition/iv/.

### Evaluation scheme

In this study, the motor imagery EEG data between 0.5 and 2.5 s (i.e. 200 sample points for dataset 1 and 2, and 500 sample points for dataset 3) after the visual cue have been extracted and used for further processing. Common average referencing is applied to the extracted raw EEG data.

Butterworth bandpass filter and SVM classifier have been used for all methods except for SBLFB where LDA is used for classification. For comparison the following experimental settings have been used for each of the methods:

- *CSP:* A bandpass filter with 7–30 Hz passband has been applied. The number of spatial filters *m* = 3 has been used.
- *CSSP:* Sample point delay *τ* in the range of 1 to 15 has been evaluated and the best value selected using 10-fold cross validation. Bandpass filter is the same as in CSP. The number of spatial filters *m* = 3 has been used.
- *FBCSP:* The experimental settings were adopted from Higashi and Tanaka [35] (as these settings gave optimal results), having 6 bandpass filters with 4–40 Hz frequency range and bandwidth of 6 Hz (no overlap). Mutual information based feature selection has been performed as it gave the best results in [32]. The number of spatial filters *m* = 3 has been used.
- *DFBCSP:* As in [36], we have used 12 bandpass filters with a bandwidth of 4 Hz in the range of 6 to 40 Hz. The number of spatial filter *m* = 1 has been used. Fisher’s ratio is used in DFBCSP (FR) and mutual information in DFBCSP (MI) for band selection, where the top 4 bands are selected.
- *SFBCSP:* 17 bandpass filters with a bandwidth of 4 Hz overlapping each other at a rate of 2 Hz was adopted from [36]. The regularization parameter λ was determined using 10-fold cross validation.
- *SBLFB:* 17 bandpass filters in the frequency range of 4–40 Hz having bandwidth of 4 Hz with an overlap of 2 Hz has been used, as used in [13]. The number of spatial filters *m* = 1 has been used.
- *Proposed approach:* 12 bandpass filters with 4–30 Hz range having bandwidth of 4 Hz with 2 Hz overlap (i.e. 4–8 Hz, 6–10 Hz, 8–12 Hz, …, 26–30 Hz) have been used. The number of spatial filters selected for these bands is *m* = 1. A 7–30 Hz wide bandpass filter is used with CSP and CSSP feature extraction. The number of spatial filter *m* = 3 has been used for the wide band. The 4 most discriminating bands are selected as we conducted several experiments on different number of bands to be selected and using 4 bands produced good results.

### Performance measures

The following performance measures have been used to evaluate the performance of the proposed method in comparison with other methods:

- Misclassification rate – the number of trials that are being incorrectly classified with respect to the entire trials.
- Cohen’s kappa coefficient (κ) – statistical method to assess the reliability of agreement between two raters. $\upkappa =\frac{p_a-{p}_e}{1-{p}_e}$, where *p*_*e*_ is the expected percentage chance of agreement and *p*_*a*_ is the actual percentage of agreement.

## Results

10 × 10-fold cross-validation is used to evaluate the performance of all experiments conducted using dataset 1, dataset 2 and dataset 3. The figure with ± represents the standard deviation.

Table 1, Table 2 and Table 3 shows the comparison of the misclassification rate of the proposed method with other competing methods in the literature. As can be seen from the results in Table 1, Table 2 and Table 3, the use of mutual information for band selection (DFBCSP-MI) shows an improved performance of 1.17%, 1.30% and 1.67% (for dataset 1, dataset 2 and dataset 3, respectively) compared to that of the original DFBCSP approach where FR is used for band selection. Our proposed method achieved the lowest average misclassification rate on all the evaluated datasets, reducing the misclassification rate by 5.15%, 2.62%, 5.82% and 5.41% (for dataset 1), 6.58%, 5.20%, 9.55% and 2.91% (for dataset 2), and 2.77%, 3.98%, 4.28% and 1.08% (for dataset 3) compared to that of CSP, DFBCSP (FR), SFBCSP and SBLFB, respectively. For 3 out of 5 subjects, 3 out of 7 subjects and 4 out of 9 subjects (for dataset 1, dataset 2 and dataset 3, respectively), our proposed method obtained the lowest misclassification rate.

Cohen’s kappa coefficient is used to further validate the reliability of the obtained results. The values obtained are given in Table 7, Table 8 and Table 9 for dataset 1, dataset 2 and dataset 3, respectively. A larger value of the kappa coefficient indicates a greater strength of agreement while a lower kappa coefficient indicates that the agreement is weak. As a rule of thumb, in [50] it is suggested that kappa coefficients in the range of <0.20, 0.21–0.40, 0.41–0.60, 0.61–0.80 and 0.81–1.0 indicate poor, fair, moderate, good and very good strengths, respectively. Highest average kappa coefficient of 0.832 for dataset 1, 0.647 for dataset 2 and 0.620 for dataset 3 are obtained by our proposed method indicating a very good strength of the prediction of classes for dataset 1 and good prediction of classes for dataset 2 and dataset 3. Subject *av.* of dataset 1, subjects *b* and *c* of dataset 2 and subjects *B0203T* and *B0303T* of dataset 3 obtained the highest misclassification rate and the lowest kappa coefficient. This may be due to the signals being contaminated by noise or due to poor recording of the signal that resulted in reducing the overall average kappa coefficient. Subjects *aa*, *al*, *aw* and *ay* of dataset 1, subjects *d* and *e* of dataset 2, and subjects *B0403T* and *B0503T* of dataset 3 obtained high kappa coefficients indicating very good strength of class prediction.

**Table 7:** Cohen’s kappa coefficient for different methods using dataset 1. The largest value for each subject is highlighted in bold

Subject	CSP	CSSP	FBCSP	DFBCSP (FR)	DFBCSP (MI)	SFBCSP	SBLFB	Proposed
*aa*	0.613	0.659	0.601	**0.816**	0.746	0.394	0.664	0.810
*al*	0.927	0.940	0.970	0.976	0.964	0.917	0.973	**0.977**
*av*	0.426	0.423	0.384	0.329	0.450	0.389	0.439	**0.540**
*aw*	0.800	0.837	0.837	0.906	0.934	0.743	0.889	**0.936**
*ay*	0.903	0.926	0.881	0.847	0.853	0.763	0.780	**0.897**
Average	0.734	0.757	0.735	0.775	0.789	0.641	0.749	**0.832**

**Table 8:** Cohen’s kappa coefficient for different methods using dataset 2. The largest value for each subject is highlighted in bold

Subject	CSP	CSSP	FBCSP	DFBCSP (FR)	DFBCSP (MI)	SFBCSP	SBLFB	Proposed
*a*	**0.736**	0.727	0.618	0.664	0.712	0.652	0.618	0.714
*b*	0.144	**0.146**	0.106	0.142	0.140	0.094	0.170	0.140
*c*	0.126	0.201	0.286	0.290	0.326	0.140	0.336	**0.380**
*d*	0.552	0.708	0.556	0.530	0.562	0.410	0.770	**0.868**
*e*	0.640	0.639	0.720	0.634	0.654	0.506	0.768	**0.838**
*f*	0.550	0.629	0.608	0.714	0.740	0.582	0.576	**0.732**
*g*	0.858	0.873	0.862	0.820	0.848	0.806	**0.882**	0.856
Average	0.515	0.560	0.537	0.542	0.569	0.456	0.589	**0.647**

**Table 9:** Cohen’s kappa coefficient for different methods using dataset 3. The largest value for each subject is highlighted in bold

Subject	CSP	CSSP	FBCSP	DFBCSP (FR)	DFBCSP (MI)	SFBCSP	SBLFB	Proposed
*B0103T*	0.526	0.494	**0.620**	0.535	0.593	0.470	0.565	0.615
*B0203T*	0.180	0.141	0.088	**0.185**	0.113	0.145	**0.185**	0.168
*B0303T*	0.008	0.031	0.018	0.010	0.073	0.100	0.014	**0.120**
*B0403T*	0.988	0.988	0.965	0.985	0.988	**0.993**	0.983	0.988
*B0503T*	0.669	0.115	0.430	0.500	0.578	0.499	**0.840**	0.810
*B0603T*	0.576	0.524	0.513	0.583	0.605	0.598	0.590	**0.640**
*B0703T*	0.718	0.724	0.690	0.758	0.805	0.755	**0.850**	0.778
*B0803T*	0.766	0.710	0.623	0.778	0.743	0.753	0.778	**0.790**
*B0903T*	0.655	0.655	0.583	0.555	**0.675**	0.500	0.613	**0.675**
Average	0.565	0.487	0.503	0.543	0.574	0.535	0.602	**0.620**

## Discussion

In the results section, we have shown that the use of mutual information for band selection gives improved results over that of using FR of single channel band power. We have also introduced a single wide band (7–30 Hz) with CSP and CSSP feature extraction in our approach. Table 4, Table 5 and Table 6 shows the top 4 bands that are mostly selected (during 10 × 10-fold cross validation) for each subject using the proposed method. The bands are not listed in any particular order of the amount of discriminant information it contains. Bands 1–12 corresponds to the 12 overlapping bands in the range of 4–30 Hz, while bands 13a and 13b corresponds to the 7–30 Hz wide band with CSP and CSSP feature extraction, respectively.

The introduced wide band is mostly selected in 4 out of 5 subjects for dataset 1, 6 out of 7 subjects for dataset 2 and 9 out of 9 subjects for dataset 3. Therefore, it is evident that introducing the wide band with CSP and CSSP feature extraction methods did play an instrumental role in improving the performance of motor imagery EEG signal classification. The selection of the wide band means the wide band have more significant features (features with larger mutual information values) that help in distinguishing between the two classes of signals. For subject *b* of dataset 2 where the wide band was not selected, it can be noted that there is no change in the misclassification rate and kappa coefficient comparing the proposed method with that of DFBCSP (MI). This is because the same bands were selected as in DFBCSP (MI), which is due to the wide band having less significant features compared to the 4 bands that were selected. On the other hand, in comparison with DFBCSP (MI), subject *d* of dataset 2 showed the largest reduction in the misclassification rate (15.30%) using the proposed method. This is mainly due to the selection of the wide band with CSSP features (13b) that contain more significant features thus contributing to the improved performance. It should be noted that Table 4, Table 5 and Table 6 only report the bands that are selected most of the time during 10 × 10 fold cross validation, and does not mean that these bands are selected all the time. This is the reason why some subjects showed improved performance using the proposed method compared to that of DFBCSP (MI) although the wide band was not selected. For example, for subject *aa* of dataset 1, improvement in the performance is noted using the proposed method compared to that of DFBCSP (MI). This is due to the facts that in some of the runs during the 10 × 10-fold cross validation, the wide bands were selected and had accounted for the improvement. However, since majority of the times the 4 bands selected for subject *aa* of dataset 1 did not include the wide band it is not shown in Table 1.

Our proposed method also outperformed the sparsity-aware and CD-CSP-FDA methods that were evaluated using dataset 1. Average misclassification rate of 12.36% and 8.92% were reported (for sparsity-aware and CD-CSP-FDA methods, respectively) while our proposed method achieved an average misclassification rate of 8.32% (an improvement of 4.04% and 0.60%, respectively) on the same dataset. The improved performance of CD-CSP-FDA was mainly due to the use of decimation filter that was manually tuned for optimal performance for each subject. The sparsity-aware method can be used for learning the spatial filters, and the decimation filter can be used for filtering the raw data in the proposed method, which may further enhance the performance of the system. Manual tuning of the filter bank is a time consuming exercise and therefore optimization algorithms should be employed to automatically tune the temporal filters.

**Fig. 4:**
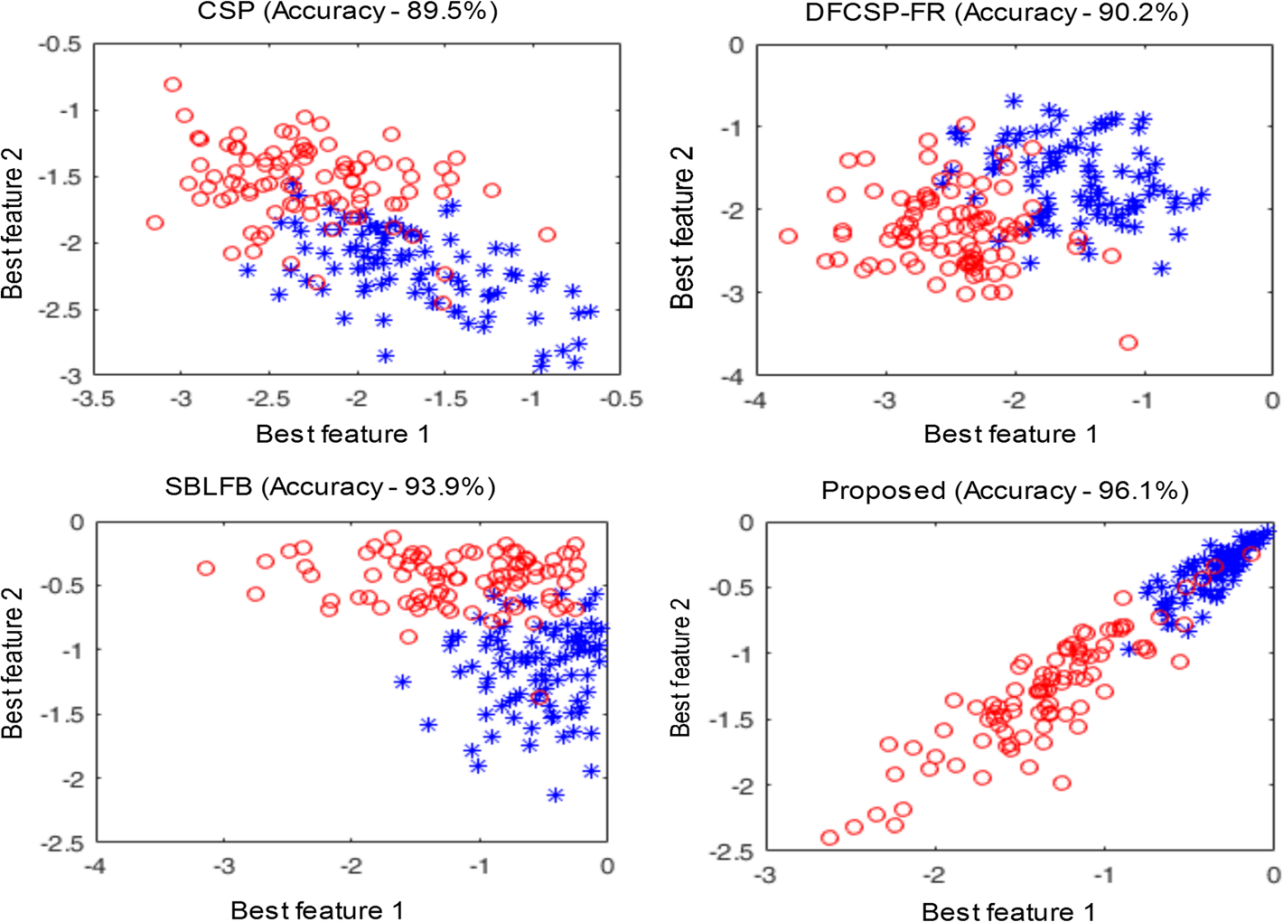
Distributions of the two most significant features of subject *d* obtained by CSP, DFBCSP (FR), SBLFB and proposed method (random experimental run), respectively

Furthermore, band selection is carried out for selecting the most discriminating filter banks that will result in more separable features for improved classification performance. The results in Fig. 4 show that our proposed method can effectively find the most separable features resulting in an improved performance in comparison with other competing methods such as CSP, DFBCSP (FR), and SBLFB. This confirms the usefulness of the proposed method. SBLFB and the proposed method attained further separable feature distributions than those of CSP and DFBCSP.

**Fig. 5:**
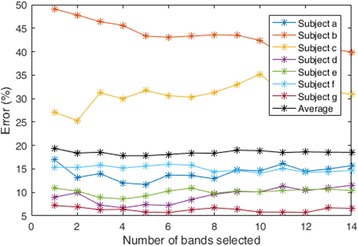
Misclassification rate for different number of bands selected (for dataset 2). Average misclassification rate using 4 bands is 17.66% and using all the bands is 18.53%

As in [36], amongst the 14 bands (the 12 overlapping bands and the wide band with CSP and CSSP features considered separately) we have selected top-r bands in the following manner. First we measured the mutual information for each of the 14 bands. Then we ranked the 14 bands according to its mutual information values. Thereafter, we selected top-r bands for which the average error rate was minimum. We found that when *r* = 4 the error rate was lowest and hence we selected 4 bands. Figure 5 shows the error rate (for dataset 2) for each of the subjects. In addition, the average error rate over all the subjects is also depicted in Fig. 5. We achieved near optimal results using r = 4 bands for dataset 1 and dataset 3 as well. Most of the subjects in dataset 2 (as shown in Fig. 5) obtained low error rate using the top 4 bands (except for subjects b and c). This suggests that selecting number of bands influences the error rate. In addition, band selection procedure also influences the computational complexity of the system.

**Fig. 6:**
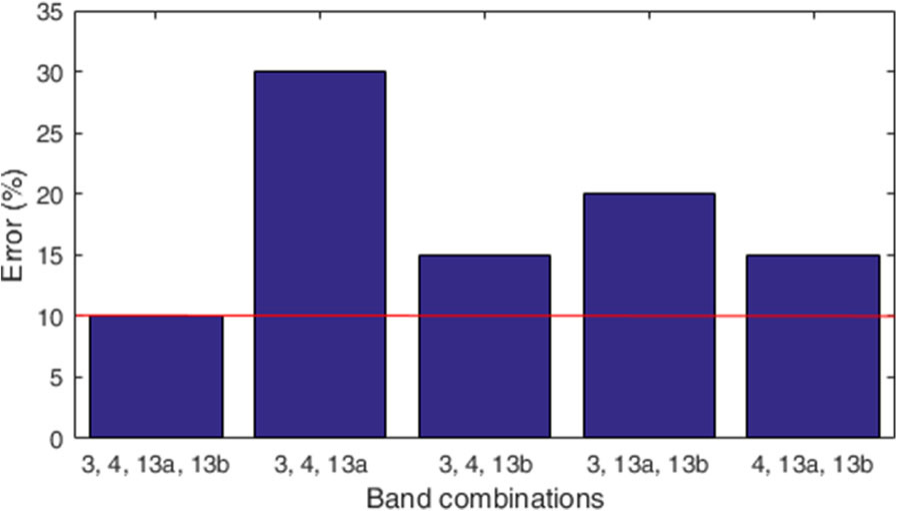
Misclassification rate (for different combinations of selected sub-bands) for one of the trial runs for subject *f* of dataset 2

To further analyse the correlation from the sub-bands, we have carried out redundancy analysis of the top 4 bands that are selected. One band is removed from the selected 4 bands and the performance in terms of the misclassification rate using the remaining 3 bands is evaluated. This procedure (of removing a band and computing misclassification error of the 3 bands) is done for all the 4 selected bands. Figure 6 shows the misclassification rates of 3 out of 4 bands for one of the trial runs of subject *f* (dataset 2). It can be observed that by removing any band (out of the 4 selected bands) increases the misclassification error rate. Particularly, the error rate increased by 20%, 5%, 10% and 5% when removing bands 13b, 13a, 4 and 3, respectively. This shows that each of the 4 bands possesses significant information and contributes towards the classification performance of the system. Removing any single band deteriorates the classification performance. Therefore, the bands do not have overlapping information or in other words are not redundant. Hence, we can say the correlations among bands are not significant by showing this redundancy analysis.

CSP has become an effective method for extracting features from motor imagery EEG signals for the purpose of classification. As a number of studies have suggested [13, 30, 32–34, 36, 40, 42, 51], sub-band optimization leads to an improved performance of motor imagery EEG based BCI systems. Accordingly, this study proposed an improved method of band selection to find most separable features utilizing the mutual information. In our work, we have introduced a wide band (7–30 Hz) to the existing overlapping sub-bands. This wide band is not the optimal wide band, however, it gives overall good results and can be adopted. For SBCSP, FBCSP, DFBCSP, SFBCSP, SBLFB, and proposed method, the bands are pre-specified i.e. determined empirically. To further improve the performance of these methods, we need to adopt the method of learning the filter band parameters (passband frequencies and cutoff frequencies) automatically. This will require the development of more sophisticated strategies to further enhance the performance of BCI systems. Furthermore, dimensionality reduction methods [52], and other feature selection methods [53] can also be studied to optimize the performance of the system using multiple-filter bands.

## Conclusions

This study introduced an improved DFBCSP method for selecting the most discriminating filter bands that would give most significant features for classification. Use of mutual information of all available channel data proved to be more effective in selecting the most significant bands compared to using FR of single channels band power as used by original DFBCSP approach. An additional wide band of 7–30 Hz has been introduced to boost the performance of the system and it is shown that the wide band effectively plays a vital part in reducing the misclassification rate. The proposed method outclassed all other state-of-the-art methods achieving the lowest misclassification rate with good overall prediction strength. Further improvements may be achieved if sophisticated algorithms are developed for automatically learning the filter band parameters.

## Abbreviations

BCI: Brain computer interface
CSP: Common spatial pattern
CSSP: Common spatio-spectral pattern
CSSSP: Common sparse spectral spatial pattern
DFBCSP: Discriminatant filter bank common spatial pattern
ECG: Electrocardiogram
EEG: Electroencephalography
EMG: Electromyogram
EOG: Electrooculogram
FBCSP: Filter bank CSP
FDA: Fishers’ discriminant analysis
FIR: Finite impulse response
FR: Fisher ratio
LDA: Linear discriminant analysis
MI: Mutual information
SBCSP: Sub-band common spatial pattern
SBLFB: Sparse Bayesian learning of filter banks
SFBCSP: Sparse filter bank CSP
SVM: Support vector machine
